# A comparison of the knowledge of hormonal contraception between women living in urban and rural areas of Poland

**DOI:** 10.1371/journal.pone.0320261

**Published:** 2025-03-28

**Authors:** Joanna Witkoś, Magdalena Hartman-Petrycka, Grzegorz Błażejewski

**Affiliations:** 1 Collegium Medicum, Department of Health Science, Andrzej Frycz Modrzewski Krakow University, Kraków, Poland; 2 Department of Basic Biomedical Science, Faculty of Pharmaceutical Sciences in Sosnowiec, Medical University of Silesia, Katowice, Poland; Delta State University, NIGERIA

## Abstract

The Contraception Policy Atlas and its latest 2024 study shows that the availability of contraception in Poland is a meagre 33.5%, the lowest of all European countries. The aim of the research, therefore, was to assess and compare the knowledge of women, living in rural and urban areas of Poland, about the modern methods of the hormonal contraception (HC) they use, includes all hormonal methods (pill, implant, patch, etc.). The study involved 239 women. The research was conducted using an online survey distributed to the public via community forums. The most commonly used method of HC was the combined pill, with 62.27% of the women surveyed using this type of contraception. None of the women surveyed used the emergency contraceptive pill. This finding is concerning given the broader context of contraceptive use in Poland. There were differences in the reasons for contraceptive use according to the place of residence. Women living in rural areas were less likely to use HC to relieve menstrual pain (28.81%) than those living in small and medium-sized towns (47.87%), and also less likely to use HC to regulate their menstrual cycle (27.12%) than those living in small and medium-sized towns (42.55%). Despite the fact that all the women who participated in this study were using HC, 13.39% stated that they did not know how this type of medication affects the female body. There was a tendency for women living in rural areas (20.34%) to be more likely to say ‘don’t know’ than the women from large towns (9.20%). The most commonly indicated side effect of contraceptives on the female body was an increased risk of thromboembolic events (70.71%).

## 1. Introduction

Around 250 million people worldwide use some form of systemic hormonal contraception (HC). However, research shows that there is still an unmet demand for contemporary methods of birth control [1]. The Contraception Policy Atlas and its latest 2024 study [2], which assesses 47 European countries in terms of access to and the financing of contraception, as well as family planning counselling and the provision of contraceptive information on the Internet, shows that the availability of contraception in Poland is a meagre 33.5%, the lowest of all European countries and, furthermore, it has remained at this low level for many years. Poland is joined at the bottom of the list by Hungary (40.0%) and Armenia (40.7%), both with a considerably higher contraceptive availability rate than Poland. By contrast, in countries such as Luxembourg, United Kingdom, France and Belgium, rates are above 90%, at 94.2%, 94.1%, 93.2% and 91.1% respectively. To improve the situation, especially in countries with the worst access to contraception, experts recommend the provision of contraceptive counselling, especially on the range of contraception available, the effectiveness of contraceptives and information on where to buy them, especially in rural areas [2]. In Poland, the probable reasons for this poor situation are limited access to gynaecologists, especially in villages and small towns, the necessity of a prescription for postcoital contraceptives, which is not in line with the European Union law, insufficient sexual education and the fact that topics related to sexual life and reproduction are regarded as taboo. Furthermore, contraception is not reimbursable for any social group in Poland, and minors must have the consent of their guardian in order to obtain this type of prescription, which may pose a problem for a teenager who wants to hide from her family the fact that she is sexually active. Research conducted by SW Research Agencies as part of the ‘Healthy She’ social programme shows that Poles are reluctant to use contraception and more than half of them do not use any method of birth control [3]. Moreover, data from the ‘Contraception for You’ programme shows that only 44.9% of patients are fully aware of how to use oral HC [4]. Special attention should also be paid to the fact that most Poles adhere to Roman Catholicism. Data from the 2021 National Census [5] show that 71.3% of the total population in Poland is Catholic, and that the principles of this religion oppose the use of contraceptive methods other than natural methods based on fertility awareness, which women are taught in premarital courses. There is therefore a combination of cultural, social and religious norms opposed to the use of contraceptives.

The unmet need for both family planning and the prevention of unplanned pregnancies remains a global public health priority. The World Health Organization (WHO), which works vigorously to promote contraception, reports that young, poorer and unmarried people are reluctant to see a gynaecologist, as are those who fear the side effects of these types of drugs. The WHO also reports that among the 1.9 billion women of reproductive age (15-49 years) living worldwide in 2021, 1.1 billion have a need for family planning. Of these, 874 million women use modern methods of contraception while 164 million have an unmet need for contraception [6].

Today, people’s reproductive behaviour is changing as it becomes more common to plan a family ‘later’ or to forgo having children altogether. The reasons for these changes range from career goals, to difficulties in finding a suitable partner, to personal values. Demographic changes observed worldwide are leading to a declining population. The global fertility rate has fallen from 3.2 live births per woman in 1990 to 2.5 in 2019 [7]. Between 2000 and 2020, the number of women using modern contraceptive methods increased from 663 million to 851 million, while the number of women planning to become mothers increased from 900 million in 2000 to almost 1.1 billion in 2021. In 2022, the worldwide use of contraception, by any method, is estimated to be 65%, with modern methods used by almost 59% of married or cohabiting women. To meet women’s expectations, the WHO is developing guidelines on the prevalence of service provision for access to modern methods of contraception as well as helping countries to implement this programme. It is also conducting implementation research to improve access to information on contraception and its safety [7]. The WHO European Action Plan calls for universal access to contraception by 2030. At the same time, the number of women using contraception is expected to increase by another 70 million [6].

Reproductive Justice [8] has established four principles: that it is a human right both to have children and to be able to prevent pregnancy through universal access to contraceptive methods, services and quality care; that sex and reproduction are not necessarily connected, as healthy sexuality and pleasure are essential components of a full human life and where a child is born, a person has the right to raise them with dignity and health in a socially supportive and safe environment. Universal access to a person’s preferred sexual and reproductive services is one of a number of human rights. The use of contraception provides the choice of either deciding not to have children at all or of deciding the number of children and the interval between births. It protects young women from unplanned pregnancies, especially those that may occur at the beginning of their education or career, and thus leads to the empowerment of women in the labour market. Consciously planned parenthood tends to involve the woman paying greater attention to her own body, thus increasing the likelihood of an undisturbed physiological pregnancy and improving the health of the woman and that of her newborn. As such, contraception and fertility control are critical to achieving the goals of sustainable economic development worldwide [8].

The Contraception Policy Atlas [2] shows that the availability of contraception in Poland is the lowest of all European countries. Therefore, the aim of the research the aim of the research, therefore, was to assess and compare the knowledge of women, living in rural and urban areas of Poland, about the modern methods of the HC they use. The authors of the study wanted to obtain information on the methods of contraception used by Polish women, their knowledge of the mechanisms of contraceptive action of these types of drugs, the possibility of side effects and the overall impact on the body. They also asked about the sources of the women’s knowledge about contraceptive methods.

## 2. Methodology

The survey targeted women who were currently using some form of HC. The sample for the study was conveniently selected with the use of a questionnaire which was distributed on internet forums that were popular among Polish women and whose administrators agreed to its publication. The questions in the survey were single- or multiple-choice, arranged so that the women could choose the answer(s) that corresponded to their knowledge and experience of contraception. The survey was structured in such a way that the women could not move on to the next question if they had not answered the previous one. Before analysing the questions, the database was checked and questionnaires from women who gave incorrect answers, e.g., anthropometric data and age outside the range of possible human values, were removed.

The participants were anonymous to the authors of the study and vice versa. There was no way of receiving written or verbal informed consent from the participants. The questionnaire was completed by volunteers who wanted to share their own experience about HC they use. The recruitment period for this study 1^st^ March 2022 – 20^th^ December 2022.

Statistical analysis was performed using the Statistica 13 program. Due to the lack of homogeneity of variance and normality of distribution, the Kruskal-Wallis ANOVA test and Chi^2^ test were used to analyse the data. Results with p < 0.05 were considered statistically significant.

This study was conducted in accordance with the Declaration of Helsinki, and approved by the Bioethical Committee of Andrzej Frycz Modrzewski Krakow University (permission number KBKA/7/O/2022).

## 3. Results

The study involved 239 women aged between 18 and 47 years, with an average age of 24.54 ±  4.76 years. Body Mass Index (BMI) ranged from 15.63 to 40.83 kg/m^2^, with a mean of 22.95 ±  4.61 kg/m^2^. Having a regular partner was reported by 220 female respondents, representing 92.05%. The frequency of sexual intercourse varied among the women interviewed: 7 women (2.93%) had intercourse daily, 105 (43.93%) several times a week, 49 (20.50%) once a week, 58 (24.27%) several times a month, 7 (2.93%) once a month, 13 (5.44%) once every few months.

Of the respondents, 59 (24.69%) women lived in rural areas, 94 (39.33%) in small and medium-sized towns (less than 100,000 inhabitants) and 86 (35.98%) in large towns (more than 100,000 inhabitants). The age and BMI of the women in the study did not differ significantly according to where they lived (Table 1). Women with secondary education predominated in the study, with a small percentage with vocational, lower secondary and basic education. Women from large towns were slightly better educated than women from rural areas, but the differences observed did not reach statistical significance. There were also no statistically significant differences in the duration of use of HC according to place of residence (Table 2). Overall, more than 60% of women had been using HC for at least a year, although most reported using it for 2-3 years. The most commonly used method of HC was the combined pill, with 62.27% of the women surveyed using this type of contraception (Table 2). Much less commonly used were the mini-pill (11.72%) and the contraceptive patch (10.04%). Other methods of HC were used far less frequently. None of the women surveyed used the emergency contraceptive pill.

**Table 1:** Age and BMI of women using HC by place of residence: rural areas N = 59, small and medium towns N = 94, large towns N = 86 (Kruskal-Wallis ANOVA ns - statistically insignificant).

	Age^ns^				BMI^ns^			
	Total	Rural areas	Small and medium towns	Large towns	Total	Rural areas	Small and medium towns	Large towns
Median	23.00	23.00	23.00	24.00	21.94	22.04	21.94	21.80
Average	24.54	24.51	23.97	25.19	22.80	22.95	22.69	22.83
Standard deviation	4.76	4.70	4.68	4.87	4.51	4.61	4.24	4.78
Minimum	17.00	17.00	18.00	18.00	15.63	16.80	15.63	16.73
Maximum	47.00	39.00	47.00	42.00	40.83	38.97	38.06	40.83

**Table 2:** Level of education of women using HC, duration of use and method of contraception by place of residence: rural areas(r) N = 59, small and medium towns (m1) N = 94, large towns (m2) N = 86; (Chi2; ns- statistically insignificant).

		Total		Rural areas (r)		Small and medium size towns (m1)		Large towns (m2)		p
		N	%	N	%	N	%	N	%	
Education	Higher	94	39.33	19	32.20	34	36.17	41	47.7	ns
	Secondary	128	53.56	36	61.02	53	56.38	39	45.3	
	Vocational, lower secondary and basic	17	7.11	4	6.78	7	7.45	6	7.0	
Length of time HC has been used	Less than one month	9	3.77	2	3.39	6	6.38	1	1.16	ns
	2-6 months	41	17.15	13	22.03	13	13.83	15	17.44	
	7-12 months	42	17.57	7	11.86	19	20.21	16	18.60	
	1-2 years	37	15.48	9	15.25	15	15.96	13	15.12	
	2-3 years	61	25.52	17	28.81	21	22.34	23	26.74	
	4-5 years	25	10.46	6	10.17	10	10.64	9	10.47	
	More than 5 years	24	10.04	5	8.47	10	10.64	9	10.47	
What type of HC are you currently using?	Combined pill	156	65.27	37	62.71	62	65.96	57	65.52	ns
	Mini-pill	28	11.72	4	6.78	16	17.02	8	9.20	ns
	Contraceptive patch	24	10.04	9	15.25	7	7.45	8	9.20	ns
	Vaginal ring	19	7.95	7	11.86	5	5.32	7	8.05	ns
	Intrauterine device (IUD)	8	3.35	1	1.69	3	3.19	4	4.60	ns
	Contraceptive implants	2	0.84	1	1.69	0	0.00	1	1.15	ns
	Contraceptive injections	1	0.42	0	0.00	1	1.06	0	0.00	ns
	Emergency contraception pill	0	0.00	0	0.00	0	0.00	0	0.00	ns

The most common reason women gave for using HCs was to prevent unwanted pregnancy (92.05%). This was followed by menstrual problems (38.08%), irregular monthly cycles (35.95%) and the desire to cure skin lesions (acne) as reported by 20.92% (Table 3). There were differences in the reasons for contraceptive use according to the place of residence. Women living in rural areas were less likely to use HC to relieve menstrual pain (28.81%) than those living in small and medium-sized towns (47.87%) (p = 0.005), and also less likely to use HC to regulate their menstrual cycle (27.12%) than those living in small and medium-sized towns (42.55%) (p = 0.054).

**Table 3:** Reasons for using HC by place of residence: rural area (r) N = 59, small and medium towns (m1) N = 94, large towns (m2) N = 86; (Chi^2^; ns- statistically insignificant).

		Total		Rural areas (r)		Small and medium towns (m1)		Large towns (m2)		
		N	%	N	%	N	%	N	%	p
^*^For what reason did you decide to use HC?	Prevent unwanted pregnancy	220	92.05	57	96.61	83	88.30	80	91.95	ns
	Menstrual problems	91	38.08	17	28.81	45	47.87	29	33.33	_r vs. m1_ = 0.005
	Irregular menstrual cycle	86	35.98	16	27.12	40	42.55	30	34.48	_r vs. m1_ = 0.054
	Acne	50	20.92	10	16.95	21	22.34	19	21.84	ns
	Doctor’s recommendation	20	8.37	3	5.08	10	10.64	7	8.05	ns
	Symptoms related to the menopause	1	0.42	0	0.00	1	1.06	0	0.00	ns
	Other reason	0	0.00	0	0.00	0	0.00	0	0.00	ns

* Question with possible multiple answers.

With regard to the level of knowledge of the women surveyed, it was found that most of them believed that modern contraceptive methods prevent pregnancy by inhibiting ovulation (88.70%), followed by thickening of the cervical mucus (65.27%) and changes in the endometrium (29.29%) (Table 4). The answer ‘endometrial mucosal changes’ was given significantly less often by women living in rural areas (20.34%) compared to women living in a town (36.78%) (p = 0.034).

**Table 4:** The women’s knowledge of the way in which hormonal agents prevent pregnancy. Responses were broken down by place of residence: rural area (r) N = 59, small and medium towns (m1) N = 94, large towns (m2) N = 86; (Chi^2^; ns-statistically insignificant).

		Total		Rural areas (r)		Small and medium towns (m1)		Large towns (m2)		
		N	%	N	%	N	%	N	%	p
^*^What do you know about how modern HC work to prevent pregnancy	Inhibition of ovulation	212	88.70	51	86.44	86	91.49	75	86.21	ns
	Thickening of cervical mucus	156	65.27	33	55.93	62	65.96	61	70.11	ns
	Changes in endometrial mucosa	70	29.29	12	20.34	26	27.66	32	36.78	_r vs. m2_ = 0.034
	Decreased contractility of the fallopian tubes	7	2.93	1	1.69	3	3.19	3	3.45	ns
	Other	0	0.00	0	0.00	0	0.00	0	0.00	ns

* Question with possible multiple answers.

Despite the fact that all the women who participated in this study were using HC, 13.39% stated that they did not know how this type of medication affects the female body (Table 5). There was a tendency for women living in rural areas (20.34%) to be more likely to say ‘don’t know’ than the women from large towns (9.20%) (p = 0.055). The most commonly indicated side effect of contraceptives on the female body was an increased risk of thromboembolic events (70.71%), followed by an increase in weight and appetite (46.86%) and an increased risk of embolism, pulmonary hypertension and endocarditis (44.77%). Compared to women living in large towns, women living in rural areas were less likely to indicate an increased risk of thromboembolic events (59.32% vs 77.01%, p = 0.022), and an increased risk of embolism, pulmonary hypertension and endocarditis (35.59% vs 51.72%, p = 0.055). Women living in rural areas were also less likely to indicate the possibility of skin discolouration due to the combination of hormone use and prolonged sun exposure (5.08% vs. 19.54%, p = 0.013) and the increased risk of endometrial and ovarian cancer (5.08% vs. 16.09%, p = 0.042).

**Table 5:** Knowledge of effects of the hormonal drugs used by respondents on the female body, by place of residence: rural areas (r) N = 59, small and medium towns (m1) N = 94, large towns (m2) N = 86; (Chi^2^; ns- statistically insignificant).

		Total		Rural areas (r)		Small and medium towns (m1)		Large towns (m2)		
		N	%	N	%	N	%	N	%	p
^*^What side effects, to your knowledge, does the use of HC have on the female body?	I know nothing about this	32	13.39	12	20.34	12	12.77	8	9.20	_r vs. m2_ = 0.055
	An increased risk of thromboembolic events	169	70.71	35	59.32	67	71.28	67	77.01	_r vs. m2_ = 0.022
	An increase in weight and appetite	112	46.86	24	40.68	49	52.13	39	44.83	ns
	An increased risk of embolism, pulmonary hypertension and endocarditis	107	44.77	21	35.59	41	43.62	45	51.72	_r vs. m2_ = 0.055
	A reduced risk of endometrial and ovarian cancer	61	25.52	12	20.34	23	24.47	26	29.89	ns
	An increased risk of developing primary liver cancer	37	15.48	7	11.86	13	13.83	17	19.54	ns
	Occurrence of irregular bleeding	35	14.64	5	8.47	13	13.83	17	19.54	ns
	The appearance of skin discolouration after exposure to the sun	30	12.55	3	5.08	10	10.64	17	19.54	_r vs. m2_ = 0.013
	An increased risk of endometrial and ovarian cancer	22	9.21	3	5.08	5	5.32	14	16.09	_r vs. m2_ = 0.042
	An increased risk of osteoporosis	18	7.53	5	8.47	5	5.32	8	9.20	ns
	A reduced risk of osteoporosis	10	4.18	1	1.69	3	3.19	6	6.90	ns
	A reduced risk of embolism, pulmonary hypertension and endocarditis	5	2.09	3	5.08	1	1.06	1	1.15	ns
	A reduced risk of thromboembolic events	5	2.09	2	3.39	2	2.13	1	1.15	ns
	A reduced risk of developing primary liver cancer	3	1.26	1	1.69	0	0.00	2	2.30	nz
	Other	0	0.00	0	0.00	0	0.00	0	0.00	nz

* Question with possible multiple answers.

The largest percentage of women reported that their source of knowledge about contraception was their doctor (85.77%). Another important source of knowledge for the women was the Internet (81.59%) (Table 6). It was observed that women living in rural areas were less likely to obtain knowledge from pharmacists than women living in small and medium-sized towns (3.39% vs. 9.20%, p = 0.050).

**Table 6:** Sources of contraceptive knowledge among women by place of residence: rural area (r) N = 59, small and medium towns (m1) N = 94, large towns (m2) N = 86; (Chi^2^; ns-statistically insignificant).

		Total		Rural area (r)		Small and medium towns (m1)		Large towns (m2)		
		N	%	N	%	N	%	N	%	p
^*^From which sources do you get your information on contraception?	Doctor	205	85.77	49	83.05	80	85.11	76	87.36	ns
	Internet	195	81.59	50	84.75	75	79.79	70	80.46	ns
	Friend/sister/mother	54	22.59	13	22.03	21	22.34	20	22.99	ns
	Pamphlets	53	22.18	13	22.03	17	18.09	23	26.44	ns
	Pharmacist	22	9.21	2	3.39	12	12.77	8	9.20	_r vs. m1_ = 0.050
	Press	7	2.93	2	3.39	1	1.06	4	4.60	ns
	Television	0	0.00	0	0.00	0	0.00	0	0.00	ns

* Question with possible multiple answers.

## 4. Discussion

A person’s reproductive rights and ability to make free choices in this most important and intimate area of life depend on many factors, including effective contraception. However, in order to incorporate birth control into a sexual life plan, a woman must have adequate knowledge of the contraceptive methods available [9]. Our own research has shown that where people live influences their knowledge of HC. People living in rural areas were less knowledgeable than those living in urban areas, although place of residence had no significant effect on the type of contraception used. The most common method of HC used by women in Poland was the combined pill. However, none of the women surveyed used the emergency contraceptive pill. D’Souza [10] synthesised the results of 508 primary studies on factors influencing contraceptive choice and their use around the world. They found that these factors are similar in many countries and include, among others, a woman’s life situation, age and education, social and cultural norms, and expectations about family size and timing. Women’s knowledge of contraceptive methods, their own beliefs, including convenience, relationship status, attractiveness of pregnancy, ease of use of a particular method of prevention, its availability, cost and previous experience are also important factors. The lack of use of the emergency contraceptive pill among the women surveyed was likely due to the fact that, without exception, all of the respondents took their prescribed contraception regularly. It is suggested that missing three or more combined pills and having intercourse may be an indication for post-coital contraception [11]. In the introduction it was mentioned that in Poland this type of pill is only available on prescription, which undoubtedly makes it difficult to obtain. It requires a visit to a gynaecologist, which may not be possible in a time-critical situation. Within the public health service there are long waiting lists for doctors’ appointments and a woman may not have the financial means for a private visit. It should also be noted that the Catholic Church has a very radical stance against post-coital contraception, which may have an impact on its use, since the use of methods that prevent the embryo from implanting in the uterus falls under the sin of abortion.

While there is no clear scientific evidence on the impact of religion on contraceptive choice and use in highly religious countries and subcultures such as Poland, there is likely to be a significant link between a country’s culture and religion and contraceptive. In a study conducted in Ireland, where the influence of religion is significant, women reported much stronger cultural than religious influences on their contraceptive choices. Young women in Ireland have been discouraged from using contraception for fear that their peers will find out they are sexually active and premarital sex is culturally unacceptable in this country. Other reasons that can stand in the way of preventing unwanted pregnancy are: stigma and secrecy about sex, sexuality and contraception, financial difficulties, or even poverty, health problems and limited control over decision-making [12].

The results of our own research found that the reason why the women surveyed used HC, regardless of where they lived, was to protect themselves from unwanted pregnancy. Other reasons for taking HC were to relieve menstrual cramps and to regulate the menstrual cycle. However, these reasons were given more frequently by women living in small and medium-sized towns than by those living in rural areas, which may indicate a lack of awareness that HC is not only used to prevent pregnancy. Although, of course, effective contraception is necessary to control fertility. Bearak et al. [13] reports that every year worldwide, 64 out of every 1,000 women aged 15-49 years will have an unplanned pregnancy, 61% of whom will want an abortion. Unplanned pregnancies are a global health problem. An estimated 40% of pregnancies worldwide are unplanned. In 2012, of 213.4 million pregnancies, 85 million were unplanned meaning that they either happened too early or were unwanted [14,15]. Such a situation undoubtedly has a negative impact on a woman’s well-being, but it is also associated with adverse economic consequences, with far-reaching implications for individuals and populations that relate to almost all of the Sustainable Development Goals. Reducing unplanned pregnancies is therefore a priority for the global public health community [7]. Unplanned pregnancies are most common among adolescent girls, both in developing and low income countries and among groups with low socio-economic status, due to limited knowledge about reproduction and sexuality and the lack of financial resources to purchase contraceptive drugs [16]. However, there are also women who become pregnant because they have the misconception that it is not possible to conceive while breastfeeding, for example. Studies have also shown that some women do not use contraception because they think the risk of pregnancy is low, they are too old, have sex too infrequently, or they believe that they or their partner is infertile [17,18]. In the United States, more than half of women who report an unplanned pregnancy do not use any form of birth control [19]. In Sweden, despite the fact that contraceptives are subsidised and contraceptive counselling is free for all women aged 25 and under, between 15% and 20% of women still have an unmet need for contraception [20].

Research consistently shows that women’s lack of knowledge about contraception and myths and fears about side effects are the main barriers to using and adhering to contraception, including daily use of the pill [10]. Our own research showed that the most commonly cited sources of contraceptive information for women were doctors and the Internet. Pharmacists were also a source of knowledge, but were more likely to be mentioned by women in small towns than those in rural areas. This is probably due to the fact that women in large towns have easier access to gynaecologists, as, firstly, there are many more of them and, secondly, the higher incomes in large towns mean that women can afford private visits to gynaecologists. In small towns, it can take several days or even months to get an appointment with a gynaecologist on the national health service, so a pharmacist with medical knowledge seems to be a competent and easily accessible source of knowledge [21]. The Internet is a widely accessible source of information that is now used by people all over the world. However, it is not a substitute for professional advice and a visit to a doctor’s clinics and therefore should not be as important a source of knowledge on health-related issues. Nonetheless, the women’s knowledge of how contraceptives work was found to be satisfactory. Most of them knew about the inhibition of ovulation (almost 90%) and the thickening of cervical mucus (about 65%). However, it was noted that women from large towns were more likely to know about changes in the endometrium than women from rural areas.

The paucity of sex education and subsequent scarcity of knowledge about contraception is the reason for the lack of control over fertility and over reproductive choice. Additionally, health services often fail to provide access to adequate information [16]. In Norway, for example, the experiences of friends and peers about contraception had a greater impact on women than advice from health professionals [22]. Women often use informal sources of knowledge, and unfortunately the knowledge gained in this way is insufficient, inaccurate or totally incorrect. Tierney et al. [1] carried out a study to determine the current level of knowledge about HC among almost 700 female students aged 18-30 from two post-secondary schools. A majority of about 70% of the young women reported current or past use of HC, while about 30% had not used such drugs. Approximately 71% of the participants in this study were aware of the possibility of cardiovascular disease associated with HC use, but only 56% of the women were aware of deep vein thrombosis. The results of this study suggest that young women are not sufficiently informed about the overall risks and benefits of HC, including thrombosis. The results also showed that participants aged 24 years and older, who were probably better educated, were more knowledgeable about thrombosis than young women who were less knowledgeable about a wide-ranging list of HC risks and benefits. Guzzo et al. [23] found that acquiring adequate and expert knowledge about contraception during adolescence is a predictor of contraceptive practice in adulthood, suggesting that adequate education of adolescents may lead to more effective and safer contraceptive practices later in life. It is also interesting to note that despite the fact that contraceptive use is part of American reproductive culture as evidenced by high rates of contraceptive use and nationwide public health efforts to further increase its use, the women there know little about contraception in a biomedical context and subsequent education campaigns related to the issue have resulted in only moderate knowledge gains. Manzer et al. [24] found that the lack of knowledge about contraception does not only relate to an ignorance of contraceptive methods, but touches on the reproductive processes which take place in the female body. Furthermore, the authors conclude that the lack of knowledge about this important aspect of human life is rooted in cultural norms that stifle and stigmatise learning about reproduction for women, and that efforts by health services to disseminate knowledge in this area may not be able to overcome these barriers.

Looking at the women’s responses to the side effects of contraceptive use in our study, the most commonly indicated adverse effect on the female body was an increased risk of thromboembolic events. However, it should be noted that women living in rural areas were less likely than those living in large towns to say that HC increases the risk of thromboembolic events, or that it increases the risk of embolism, pulmonary hypertension, endocarditis, darkens the skin after prolonged sun exposure, and increases the risk of endometrial and ovarian cancer. When the first HC, the contraceptive pill, appeared in the 1960s, there was controversy over its side effects. The pill played an extremely important role in changing women’s lives. It allowed them to have fewer children, experience better health and have more opportunities for education and work [25]. Despite the many undisputed advantages, there were also disadvantages to the use of these drugs in the form of the risk of cancer, or the onset of cardiovascular disease. Although the composition of modern contraceptive drugs has changed over the years, the risk of adverse side effects is still a major concern, causing many women to choose not to use, or to discontinue, modern contraceptive methods. Studies have shown, however, that it is the psychological and sexual side effects that are the most common reason why women have stopped using contraception and have not followed medical advice, rather than the risk, for example, of thromboembolic events [26–29].

Assistance and advice from health professionals on contraceptive choice is essential, however, it should be noted that the media and social media forums now have a strong and direct influence on women’s contraceptive decisions. In particular, the opinions of influencers on whether or not to use contraception can have a significant impact on the decisions of young women. For example, it was noted that influencers in Sweden often recommend natural family planning instead of HC. Digital fertility apps are recommended and positively promoted as ‘hormone-free’ alternatives on social media [30,31]. Over the past decade, women in Western countries have begun to make extensive use of various social media platforms to share their unsatisfactory experiences of contraception. However, these ‘online references’ have been widely condemned for spreading ‘misinformation and panic’ and exacerbating ‘hormoneophobia’. Related to this is the current phenomenon and clearly growing trend in Western societies towards a ‘return to nature’ and ecology. The demand for greater naturalness has also emerged in the field of reproductive health [32,33]. There is scientific evidence to suggest that there are groups of women who consider contraception to be unnatural, alien and invasive In Sweden, for example, the ‘fear of hormones’ increased significantly between 2013 and 2017, which was the reason for the massive non-use of HC. Women reported negative perceptions of oral contraception. They cited adverse mood changes and the fear of infertility, thrombosis and weight gain as the main reasons for discontinuing the combined pill [20].

To promote health and pre-conception care Moos et al. [34] developed a tool called Reproductive Life Plan (RLP). The tool helps to implement effective family planning strategies and avoid unwanted pregnancies and adverse health effects that could affect future fertility. The implementation of RLP has proven to be an effective tool in increasing women’s knowledge of fertility and their awareness of factors affecting pre-conception health. Research by D’Souza et al. [7] is based on 50 systematic reviews of 877 primary studies on interventions to support the choice and use of contraception from 72 countries. It found that environmental interventions, motivational interviewing, contraceptive counselling and psychosocial interventions had the strongest impact on contraceptive use. This was followed by school education and information provided through mobile phones and mass media [7].

Access to health services and professionals affects both knowledge about and the worldwide use of contraceptives. Health promotion, properly integrated into public policy combining sex education with the promotion of effective contraceptive methods, would certainly provide guidance for a later sexual life, especially for young women. Accurate and comprehensive information about the possible risks and side-effects associated with the use of modern contraceptive methods would dispel existing myths and misinformation, and this would certainly reduce the number of unplanned pregnancies, especially among teenagers [10,35,36]. Clinicians should facilitate women’s access to contraceptives by assisting in their choice and use. Telemedicine access to doctors, prescriptions for a year’s supply of pills and the avoidance of frequent visits to the doctor’s clinics would help women to feel more ‘cared for’ in terms of contraceptive control. This seems to be a necessary condition for women to make informed choices about controlling their own fertility. There are a number of steps and processes involved in accessing and accepting an appropriate method of contraception, and then using it correctly and consistently to avoid unplanned pregnancy. Clearly, better provision of health services in this area of medicine and the training of providers of such services can be effective, but these services must take into account the complex web of social, cultural and religious factors that facilitate or inhibit the use of contraception in a given society [10,35,36]. Birth control interventions typically focus on the woman’s knowledge and decision-making, however, it is important to remember that the attitudes and expectations of partners, family, peers and wider society have a powerful and sometimes direct influence on this aspect of a woman’s life.

The lack of knowledge about contraception in Poland, demonstrated both in the 2024 Contraception Policy Atlas [2], as well as in our own research, points to the need for urgent educational measures to raise the level of awareness of sexual life and methods of preventing pregnancy among the Polish population. The medical community (gynaecologists, sexologists, perinatologists) in Poland were so concerned with the data from the Atlas [2] that, as a result, the ‘Right to Contraception’ coalition was established. This coalition was formed by health professionals and an non-governmental organization (NGO) representatives as a response to the severely restricted access to contraception in Poland. In October 2024, a declaration was signed advocating free contraception up to the age of 25 and the establishment of a network of counselling centres focusing on sexual education and dedicated contraceptive counselling. It was agreed that the findings of the meetings should be submitted to both the Ministry of Health and the Prime Minister’s Office.

In 2024, after a change in government, there was a public debate in Poland about easier access to the emergency contraceptive pill. Since the beginning of May 2024, an emergency contraceptive pill pilot programme, commissioned by the National Health Service has been operating in Poland. Unfortunately, in those pharmacies selling the emergency contraceptive pill, which is only 1,300 out of a total 12,000 pharmacies, the pharmacists require parental consent for underage patients to purchase the pill. From the start of the pilot to December 2024, pharmacists wrote 10,830 prescriptions for the emergency contraceptive pill. Patients could make use of the pilot scheme once a month, and pharmacies were most often approached by women aged 20-29 years old, for whom 4,031 prescriptions were issued. Teenagers aged 15-19 were much less likely to contact pharmacies, with only 1,635 prescriptions written for them.

In January 2025, the Polish Government responded with a draft amendment to the Pharmaceutical Law regarding the availability of the emergency contraceptive pill without a prescription for women over the age of 15. This will provide access to contraception for young women, something which is very commonly available in European Union countries, but which has hitherto been virtually unavailable in Poland. The government is additionally working on a law that will allow 15, 16 and 17-year-olds to not only visit a gynaecologist without a parent or legal guardian but also allow them give their own consent to any medical procedures.

The Foundation for Women and Family Planning (FEDERA), an NGO fighting for reproductive justice, also responded to the problems of access to contraception by opening its own health centre in Warsaw a few months ago. This centre provides sex education and access to gynaecological, sexual and psychological care. Such an initiative is undoubtedly important, but this type of resource should be extended to the whole country, especially with regard to public health initiatives in rural areas, where access to a specialist gynaecologist or sexologist is difficult, if not impossible.

## Limitations

This study focused on assessing the knowledge of birth control methods among women living in Poland and their use of HC. Only women were included in the study, and the opinions of couples living together or wider socio-cultural-religious issues that undoubtedly influence contraceptive use and birth control were not taken into account. The authors of the study did not assess what proportion of women in Poland do not use any HC at all. There is also no known scientific evidence in Poland on the influence of religion on the decision to use contraception or to abstain from using methods other than the so-called ‘marriage calendar’ (rhythm method), the principles of which are taught in Catholic premarital courses. Differences in knowledge about the use of HC between rural and urban dwellers have been shown, but this research has not identified the main reasons for these differences. It can only be surmised that this may be related to differences in education levels, access to gynaecologists and pharmacists, as well as social and religious influences, rather than a difference in decisions made by the women in these different places of residence. The reasons for lower contraceptive knowledge in rural areas require further independent research.

## 5. Conclusions

Living in a Polish village or town did not significantly influence the choice of a hormonal contraceptive method among women using this type of method. However, living in a rural area did have a significant effect on women’s knowledge about contraception.

The most common method of fertility control chosen was the combined hormonal pill. None of the women surveyed used the emergency contraceptive pill. The main reason for using HC, regardless of place of residence, was to protect against unwanted pregnancy. The most important sources of knowledge about modern methods of contraception for the women surveyed were their doctor and the Internet. On the basis of their knowledge, respondents were able to answer that the mechanism that prevents pregnancy with hormonal contraceptives is the inhibition of ovulation and the thickening of cervical mucus. Women from large towns were more likely to report changes in the endometrium than women from rural areas. The most commonly reported side effect of contraceptives was an increased risk of thromboembolic complications.
